# Segmental Mobility, Interfacial Polymer, Crystallization and Conductivity Study in Polylactides Filled with Hybrid Lignin-CNT Particles

**DOI:** 10.3390/nano15090660

**Published:** 2025-04-26

**Authors:** Panagiotis A. Klonos, Rafail O. Ioannidis, Andreas Pitsavas, Nikolaos D. Bikiaris, Sofia P. Makri, Stefania Koutsourea, Alexios Grigoropoulos, Ioanna Deligkiozi, Alexandros Zoikis-Karathanasis, Apostolos Kyritsis, Dimitrios N. Bikiaris

**Affiliations:** 1 Dielectrics Research Group, Department of Physics, National Technical University of Athens, Zografou Campus, GR-15780 Athens, Greece; akyrits@central.ntua.gr; 2 Laboratory of Polymer Chemistry and Technology, Department of Chemistry, Aristotle University of Thessaloniki, GR-54124 Thessaloniki, Greece; rafailio@chem.auth.gr (R.O.I.); apitsava@chem.auth.gr (A.P.); nikompik@pharm.auth.gr (N.D.B.); dbic@chem.auth.gr (D.N.B.); 3 Creative Nano PC, 43 Tatoiou, Metamorfosi, GR-14451 Athens, Greece; s.makri@creativenano.gr (S.P.M.); s.koutsourea@creativenano.gr (S.K.); a.grigoropoulos@creativenano.gr (A.G.); a.karathanasis@creativenano.gr (A.Z.-K.); 4 AXIA Innovation GmbH, Fritz-Hommel-Weg 4, 80805 München, Germany; ide@axia-innovation.com

**Keywords:** polylactide, hybrid lignin–CNT, segmental mobility, electrical conductivity, rigid amorphous fraction

## Abstract

A newly developed series of polylactide (PLA)-based composites filled with hybrid lignin–carbon nanotube (CNTs) particles were studied using thermal and dielectric techniques. The low CNT content (up to 3 wt%) aimed to create conductive networks while enhancing particle–polymer adhesion. For comparison, PLA composites based on lignin and CNTs were also examined. Although infrared spectroscopy showed no significant interactions, calorimetry and dielectric spectroscopy revealed a rigid interfacial PLA layer exhibiting restricted mobility. The interfacial polymer amount was found to increase monotonically with the particle content. The hybrid-filled PLA composites exhibited electrical conductivity, whereas PLA/Lignin and PLA/CNTs remained insulators. The result was indicative of a synergistic effect between lignin and CNTs, leading to lowering of the percolation threshold to 3 wt%, being almost ideal for sustainable conductive printing inks. Despite the addition of lignin and CNTs at different loadings, the glass transition temperature of PLA (60 °C) decreased slightly (softer composites) by 1–2 K in the composites, while the melting temperature remained stable at ~175 °C, favoring efficient processing. Regarding crystallization, which is typically slow in PLA, the hybrid lignin/CNT particles promoted crystal nucleation without increasing the total crystallizable fraction. Overall, these findings highlight the potential of eco-friendly conductive PLA composites for new-generation applications, such as printed electronics.

## 1. Introduction

Poly (lactic acid), or else, polylactide (PLA), is a vastly studied aliphatic polyester that has found use in various applications, from the everyday life to academia and industry [1,2,3]. PLA, like many other known polymers, combines a superior performance, effectiveness and economic processing [4,5] with sustainability, serving as a ‘green polymer’ [6,7]. The green character of PLA owes to the potential for its synthesis from renewable resources, such as corn starch, beet or sugar cane and potatoes [8,9], in addition to being relatively non-toxic or else biocompatible [10]. Thus, PLA has been exploited as a key polymer in many applications, for example, in packaging [10,11,12], in 3D-printing devices [13,14,15] and in bioengineering and biomedicine [16,17,18].

PLA demonstrates high thermal and chemical stability [19] and, simultaneously, relatively low glass transition temperatures, *T*_g_ ~50–60 °C, and melting temperatures, *T*_m_ ~160–190 °C [1,20,21,22,23]. The latter facts set the processing of this polymer as relatively effective, mild and economically beneficial, always, compared to other known industrial polymers synthesized employing fossil-based resources (polyethylene, polystyrene, polyαcrylates, etc.). The second major beneficial property of PLA is its compatibility with many other polymers, both green and not. There are numerous studies in the literature that prove the successful combination of PLA with other polymers in the form of copolymers, interpenetrating polymer networks and polymeric mixtures [3,23,24]. Next to these aspects, the semicrystalline character of PLA is of major importance. The degree of crystallinity and the semicrystalline morphology (size, distribution and interconnectivity of the crystals) of this polyester can be quite easily tuned to a wide extent. The tuning can be achieved either by the ab initio structure of PLA, namely, the low-high molar mass and the L/D -lactide stereoisomeric ratio, as well as by thermal processing [22,25,26] and the use of additives (e.g., nano-inclusions) [27,28]. The aspects of crystallinity are of severe importance, as they are tightly connected to both the macro- and nano-scopic performance of the materials, e.g., mechanical [27,29], the *T*_g_ [21,22], small molecules permeation [30,31], heat transport [32,33] and even compostability [34,35]. An additional crucial aspect regarding polymers and polymer nanocomposites is the electrical conductivity. While most of the known polymers are insulators, due to the absence of free charges (electrons), the dispersion of conducting particles to the polymer matrix may lead to the formation of continuous conducting paths throughout the nanocomposite matrix (percolation theory) [36]. Such particles can be, for example, the carbon nanotubes, graphene platelets or metallic nanoparticles (Ag, Cu, etc) [37,38]. This is actually one of the goals of the present study, i.e., the application of polymer nanocomposites within printing inks applications. Among others, the manipulation of the conductivity of the nanocomposite provides additional potential for applications, for example, in health monitoring and sensors [39,40,41,42].

Regarding polymer composites and nanocomposites, the most widely employed reinforcing means are inorganic particles of various geometries, namely, spherical particles (e.g., silica), nano-sheets and nano-clays (e.g., graphene and montmorillonite) and nanotubes (e.g., carbon nanotubes). In the modern economic frame of green and circular economy, we witness a shift toward the development of completely green and renewable composites [43,44,45,46]. Lignin is one among the most promising candidates for replacing the conventional fillers. Lignin is usually addressed as soda- or kraft-lignin [47]. It is an aromatic biopolymer of, however, relatively high *T*_g_ (*T*_g_ >100 °C) [48], which can be isolated from bio-resources, in particular, lignocellulosic biomass. After cellulose, lignin is considered to be the second most abundant natural polymer [49,50], demonstrating the combination of low toxicity with high biocompatibility. It exhibits a variation of functional groups and occupies high carbon amounts, with the latter being potentially transformed into composites and carbon-rich materials [47]. The addition of lignin in PLA (and other polyesters) has been proven to be a good means to enhance the antioxidation and antibacterial performance [51,52,53,54]. Despite the extensive amount of literature on neat PLA and PLA filled with conventional nano- and micro- particles, less attention has been paid to biobased polymer composites. Furthermore, there is still much space for studies focusing on lignin-filled PLA composites [54,55,56,57].

In the present work, we have prepared a series of PLA-based composites, within which lignin was the main reinforcing means, via simple methods (solutions’ casting and mixing). In particular, we added hybrid particles containing 90 wt% soda lignin and 10 wt% multiwalled carbon nanotubes (MWCNTs and CNTs, for the sake of simplicity). The amount of hybrid lignin–CNT particles varied between 0.5 and 30 wt%. Following the exact preparation method, we also prepared a short series of PLA/Lignin and PLA/CNT composites, to be studied comparatively with the PLA/hybrid. Among the structure–properties relationships being investigated here, by known methods for examining structure, interactions, thermal transitions and molecular mobility, we studied the electrical conductivity of the systems. Among others, we aimed to investigate whether an interfacial polymer layer in the composites is formed in addition to the formation of electronic conducting paths (via CNTs) throughout the PLA matrix. This is because these ‘green composites’ are envisaged as conductive fillers in inks, within which the existence and potential manipulation of electrical conductivity is wanted in order to manufacture printed electronics. To directly assess the impact of the hybrid particles with those of neat lignin and neat CNTs, the conductivity results were also compared with those of neat CNTs at similar amounts. Since PLA is semicrystalline, in order to assess the direct effects of the fillers on the various properties (i.e., glass transition, segmental dynamics, electrical conductivity), the main study involved the materials in the amorphous state, whereas the crystallinity aspects are studied secondly.

## 2. Materials and Methods

### 2.1. Materials

The materials of the main investigation are composites based on PLA filled with 0.5–30 wt% lignin–CNT hybrid particles. The hybrid materials consist of soda lignin/CNT with a 90/10 ratio (wt/wt), synthesized by Creative Nano PC (Athens, Greece). Hybrids were synthesized via a patented ultrasound-assisted method [58]. Soda lignin (Protobind 1000) was purchased from Tanovis AG (Rüschlikon, Switzerland) and MWCNTs with >96% purity and outside diameter 8–18 nm were purchased from Nanografi (Ankara, Turkey), whereas the Luminy^®^ PLA L175 of melt flow index (MFI) at 8 g/10 min (Flow, 210 °C/2.16 kg) and 3 g/10 min (Flow, 190 °C/2.16 kg) was purchased from Corbion N.V. (Gorinchem, The Netherlands).

Briefly, masterbatches of PLA/hybrid were prepared by solution casting, in particular, of PLA/chloroform at 10% *w*/*v* and, subsequently, melt-mixing. First, the hybrid additives at wanted mass amounts were diluted in chloroform at 3% *w*/*v* and subjected to ultra-sonication. The said solutions were mixed and mechanically stirred. The composites were allowed to dry overnight, so that the excess chloroform was evaporated. Finally, the dried masterbatches and dried PLA were added to a melt-mixer, Haake–Buchler twin screw co-rotating extruder, and a mixing head with a volumetric capacity of 11 cm^3^, operating at 190 °C and 30 rpm for 5 min. In total, seven PLA/Hybrid systems were prepared. For comparison of the hybrid particles’ performance, we have also prepared and parallelly studied PLA-based systems filled with either neat lignin or neat CNTs at the loadings of 0.5, 1.0 and 2.5 wt%, via similar routes (i.e., three PLA/Lignin and three PLA/CNT samples).

The samples were mainly studied in the form of disks of ~1 mm in height (thickness) and ~20 mm in diameter, prepared by melt-pressing employing homemade molds.

### 2.2. Infrared Spectroscopy

Attenuated total reflection Fourier transform infrared (ATR-FTIR) spectra were recorded on amorphous samples. The measurements were performed employing an IRTracer-100 spectrophotometer by Shimadzu (Kyoto, Japan) equipped with a QATR™ 10 Single-Reflection ATR Accessory with a Diamond Crystal. The spectra were recorded in absorbance mode, within the wavenumber region from 400 to 4.000 cm^−1^ at steps of 2 cm^−1^. The presented spectra correspond to a total of 32 co-added scans, which were normalized and baseline corrected.

### 2.3. Scanning Electron Microscopy (SEM)

To examine the micromorphology and filler distribution in the polymer matrix, we performed SEM measurements, by means of an emission scanning electron microscope (JEOL (Tokyo, Japan) JSM 7610F) operating at 5 kV. The SEM images were captured at the materials’ cryo-fractured cross-sections, upon gold sputtering.

### 2.4. Differential Scanning Calorimetry (DSC)

Conventional calorimetry was employed for the study of the polymeric thermal transitions within the temperature range from –10 to 200 °C in a high-purity nitrogen atmosphere. A TA Q200 DSC calorimeter (TA Instruments, New Castle, DE, USA) was employed for the thermal study, whereas the measurements were carried out on samples of ~7–9 mg in mass closed in aluminium standard TA pans. Prior to the measurements, the calorimeter had been properly calibrated for the temperature and enthalpy, using indium, and for the heat capacity, using sapphires.

A first heating scan from room temperature up to 200 °C was performed for all samples, in order to erase the thermal history and optimize the thermal contact between the sample and the pan. Then, two main scans were conducted, as follows. The first scan involved a fast cooling at non constant rates (i.e., 60–100 K/min), while the second scan involved a slower cooling at 20 K/min. The subsequent heating for both scans was carried out at the fixed heating rate of 10 K/min.

### 2.5. Broadband Dielectric Spectroscopy (BDS)

The electrical conductivity at RT~20 °C was evaluated by dielectric spectroscopy [59] and by means of Novocontrol BDS setup (Novocontrol GmbH, Montabaur, Germany) on sample in the form of thin disks (~1 mm in thickness and 14–15 mm in diameter). Upon application of an alternate to the sample capacitor, the complex dielectric permittivity, ε* = *ε*′ − *i*∙*ε*″, was recorded in the frequency, *f*, range from 10^−1^ to 10^6^ Hz. The *f* dependence of complex electrical conductivity, σ*, was estimated from ε* via Equation (1):

(1) $$\sigma^{*}\left(\omega \right)=i*\omega *\varepsilon_{o}*\varepsilon^{*}(\omega )$$

wherein *ω* is the angular frequency (*ω* = 2π∙*f*) and *ε*_0_ is the dielectric permittivity of the vacuum. The real part of σ* is taken as the AC conductivity, *σ*′.

Additionally, the segmental dynamics were studied by BDS by following the imaginary part of the dielectric permittivity, *ε*″(*f*), at various temperatures from 30 up to 120 °C. *ε*″(*f*) was isothermally recorded at steps of 5 and 10 K. We recall that the samples had been kept initially in the amorphous state by melting and fast cooling to room temperature (RT < *T*_g_).

## 3. Results and Discussion

### 3.1. Structure—Dispersion of Particles

**Figure 1:**
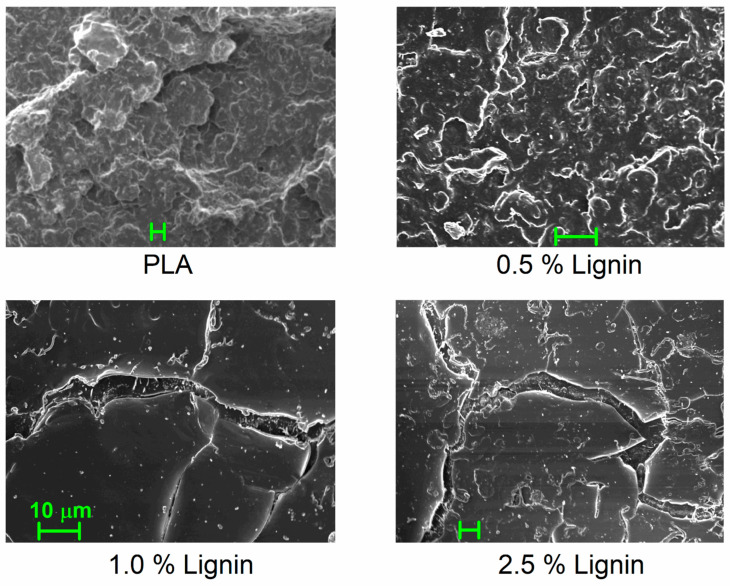
SEM images for neat PLA and PLA/lignin composites. The added scale bar corresponds to 10 μm for all images.

Before presenting the results on the thermal transitions and molecular mobility, it is essential to provide a microscopic view of the studied samples. In Figure 1, we present selected SEM images for neat PLA and the PLA/lignin composites. Lignin seems to be very well dispersed throughout the PLA matrix, forming entities with sizes below 1 μm, in general. Obviously, larger lignin aggregates co-exist (up to 4–5 μm), especially for the composites with the larger loadings.

**Figure 2:**
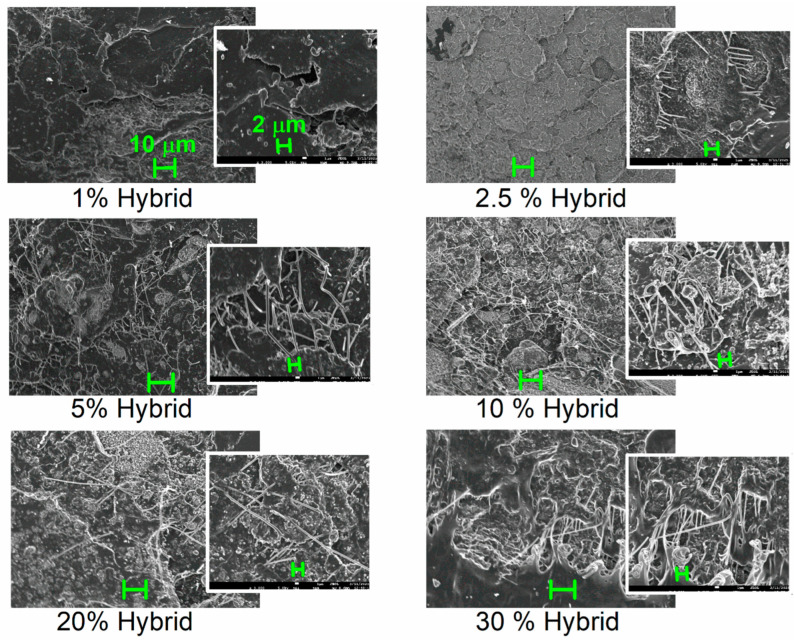
SEM images for all PLA/hybrid composites. The scale bars added to the main images correspond to 10 μm, whereas those added to the insets correspond to 2 μm.

In Figure 2, we show SEM micrographs for the PLA/hybrid composites with loadings from 1 to 30% and at two magnifications (main and inset images). The hybrid particles dispersion is quite good throughout the polymer matrix. We would like to report that the shown images are actual representatives of the overall composite volume, not of a selection of the best recorded dispersions. The existence of the hybrid lignin–CNT entities can be seen within the images for loadings of 5–30%. The presence of CNTs is clear for all cases, whereas it is interesting that the dispersion of the CNTs themselves is quite good as well. Especially for the higher loadings of 10–30% hybrid, or else, 1–3% CNTs, some continuous CNT–CNT paths have been formed. This is expected to affect a series of macroscopic properties, in particular, the electrical conductivity, the latter being studied in a later section.

### 3.2. Glass Transition and Interfacial Adhesion

**Figure 3:**
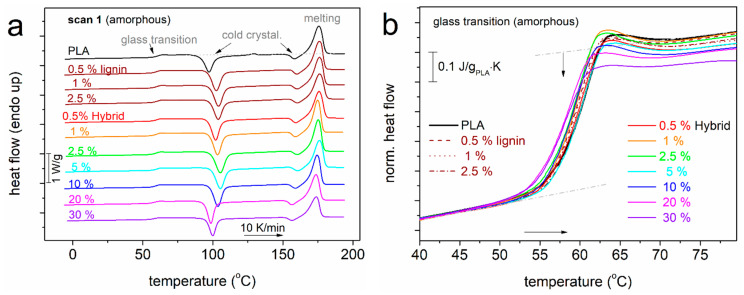
(**a**) Comparative DSC heating traces of scan 1. The shown heat flow is shown upon normalization to the sample mass (W/g). Indicated are the main thermal transitions recorded. (**b**) Details of the glass transition region, shown upon normalization of the recorded heat flow to the polymer mass and in *c*_p_ units (J/g_PLA_∙K).

Following are the results by calorimetry for scan 1. During the cooling scan at relatively fast rates, no exothermal event (peak) related to crystallization is recorded. Therefore, we consider the polymers amorphous at the beginning of the subsequent heating (Figure 3), at least up to temperatures closely above the glass transition. The main values of interest can be found in Table 1 and in the following figures. Within the temperature range studied and in the order of increasing temperature, single glass transition steps were recorded, as were cold crystallizations (2 peaks) and single melting events. Considering the numerous published findings for PLA [21,25,60,61], we conclude that all the thermal events originate from PLA.

**Figure 4:**
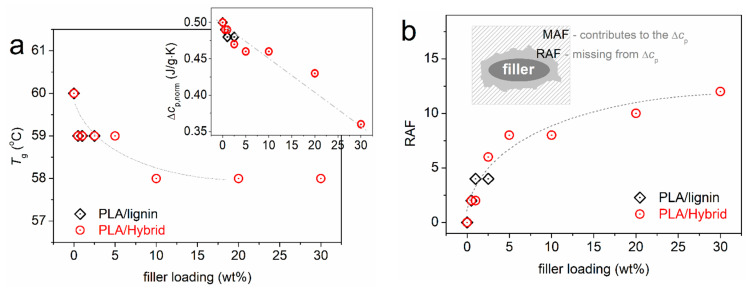
The filler content dependences of (**a**) glass transition temperature and heat capacity change (inset to (**a**)) and (**b**) the calculated rigid amorphous fraction (interfacial polymer) for all systems. Please note that the data refer to the polymers in the amorphous state. The added lines are simple eye guides. The inset to (**b**) presents a simplified schematic for rationalizing the existence of RAF in the polymer/filler composites.

Since we have preserved the amorphous character of PLA, we may seek for the direct effects of the hybrid particles on PLA. The glass transition offers this opportunity. In Figure 3b, we have focused on the glass transition, moreover, upon normalization of the thermograms to the mass of PLA and presenting the data in heat capacity, c_p_, units. In addition, we vertically translated the heating traces, so that the low temperature-side baselines coincide for all samples. Regarding the temperature position of the glass transition step, a slight *T*_g_ decrease is observed with the addition of particles, both hybrid and neat lignin. The characteristic *T*_g_ values are presented in Figure 4a, showing a weak drop from 60 °C (neat PLA) by 2 K in the composites. Moderate effects on the *T*_g_ have been presented in the past in PLA/CNT [62] and in PLA/CNT–lignin systems [63].

The most striking effect on glass transition is recorded on the strength of the transition, i.e., on the heat capacity change, Δc_p_ (vertical arrow in Figure 3b). In the inset to Figure 4a, the Δc_p_ of PLA (0.50 ± 0.1 J/g·K) exhibits a monotonic suppression with the filler addition. Since we have no other parameters implementing, e.g., a molecular weight change, the said suppression arises from the presence of the filler. In many previous works on polymer nanocomposites [61,64,65,66,67], the Δc_p_ suppression has been connected to the presence of a formed interfacial polymer layer onto the fillers’ surfaces. This layer is believed to extend to a few nanometers—from the filler surface and to exhibit either an immobilization [64,65] or quite retarded mobility [66,68,69,70]. When being immobile, or else rigid, this polymer fraction obviously does not directly contribute to the glass transition. Therefore, a missing in Δc_p_ is recorded. This rigid amorphous fraction, RAF, has been initially studied by Wunderlich [71], and a mathematical method for estimating its amount, based on Δc_p_, has been proposed. That is expressed mathematically via Equation (2):

(2) RAF=1−∆cp,norm∆cp,matrix×100%

wherein Δ*c_p_*_,*norm*_ corresponds to the composite and Δ*c_p_*_,*matrix*_ is the heat capacity change of the neat amorphous PLA.

The estimated RAF is listed in Table 1 and shown in Figure 4b. RAF increases monotonically with increasing the hybrid content from 2 to 12%; however, this is not linear [64,65,72]. The latter suggests that, most probably, the distribution of the hybrid particles is better for the lower contents and becomes worse for the higher ones [73], which is more or less the main situation for composites prepared by simple mixing [70]. However, we recall that from the SEM data (Figure 1 and Figure 2), the dispersion of the fillers is quite good in all cases.

The formation of the interfacial rigid polymer layer is generally considered to be due to strong attractive interactions between the fillers and the neighboring polymer chains [69,73]. Regarding polyesters, such as PLA here, the interaction can be evidenced by a disturbance in the vibration of the ester bond (-C=O), located at their most polar site (carbonyl). The mentioned disturbance has been recorded, for example, by infra-red spectroscopy, as either an increasing contribution of the radiation absorbance at lower wavenumbers or as an overall migration of the absorbance peak toward lower wavenumbers.

**Figure 5:**
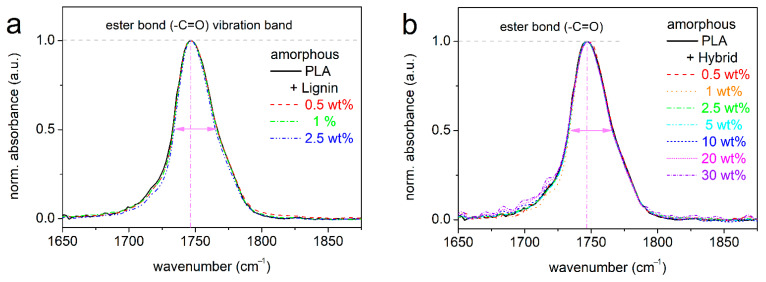
Comparative FTIR spectra focusing on the wavenumber band of absorbance arising from the vibration of the ester bond of PLA, shown for (**a**) PLA and PLA/lignin and (**b**) PLA and PLA/hybrid, all polymers being in the amorphous state. The absorbance peaks are shown upon shape normalization to the peak maxima.

Following previous methodologies, we present, in Figure 5, the FTIR spectra within the ester bond vibration band for PLA/lignin (Figure 5a) and PLA/hybrid (Figure 5b), with all systems being in the amorphous state. In none of the composites did we record such worth-noting disturbance of the ester bond peak. Therefore, we could conclude that the presence of RAF here could not be due to formed interfacial bonding, at least, not uniquely or to a vast extent, but to topological constraints imposed on the PLA chains. In this context, we recall that the composites preparation involved solution castings and mixings, thus implementing polymer chains with maximized degrees of motion freedom [74]. This could facilitate the entanglements between hybrid particles (CNTs) and the PLA chains to a high level [75]. Such entanglements can be also facilitated by the increase in the filler’s specific surface area/surface roughness [66] and aspect ratio [28,33,76]. These are expected to increase with the involvement of CNTs, individually and in combination with lignin in the hybrids.

### 3.3. Effects on the Polymer Crystallization

**Figure 6:**
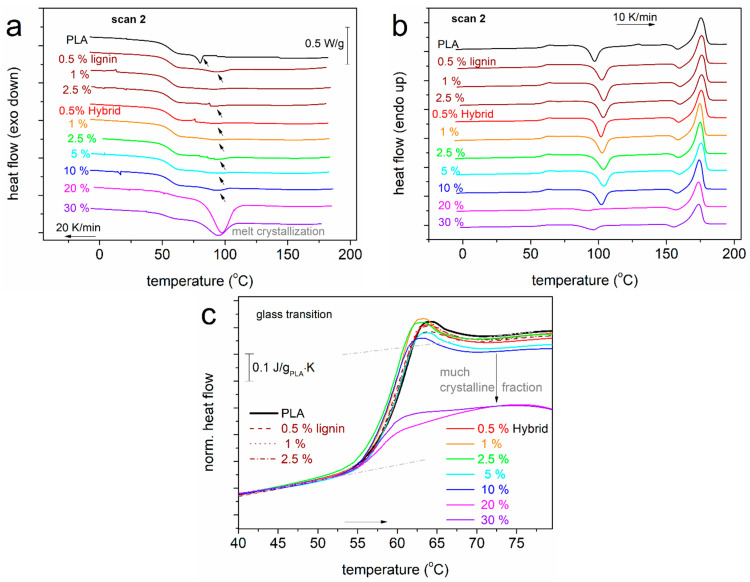
Comparative DSC traces for scan 2, namely, (**a**) during cooling from the melt state at 20 K/min and (**b**) during the subsequent heating at 10 K/min. The heat flows in (**a**,**b**) are shown upon normalization to the sample mass. (**c**) Details in the glass transition region for all samples, upon normalization of the heat flow to the polymer mass and in presented in c_p_ units.

In this section, we discuss the effects imposed by the presence of lignin and hybrid particles on the crystallizability of PLA. In Figure 6, we present the DSC thermograms for scan 2, i.e., upon erasing the thermal history, cooling the samples at a moderate rate (20 K/min, Figure 6a) and a subsequent heating (10 K/min, Figure 6b). During cooling, a weak exothermal peak is recorded in neat PLA at T_c_ = 80 °C (Table 1), corresponding to the melt/hot crystallization. By recording the corresponding enthalpy change (ΔH_c_) and comparing it with the bibliographic value for the 100% crystalline PLA (~93 J/g) [77], we calculated the crystalline fraction as CF_c_ = 1 wt%. The melt crystallization is very low also for the PLA/lignin and most PLA/hybrid systems. The exception to that is the case of 20% and 30% hybrid, within which the crystallization is strongly enhanced.

**Figure 7:**
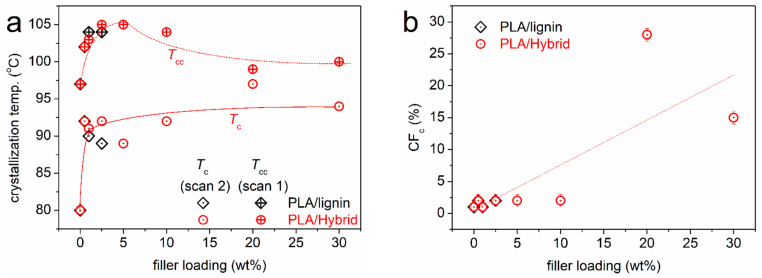
(**a**) The filler content dependences of T_c_ and T_cc_, from scans 2 and 1, respectively. The added lines are used as guides for the eyes. (**b**) The corresponding dependence of the calculated crystalline fraction during melt crystallization, CF_c_. The added line corresponds to the linear fitting to all data.

The T_c_ and CF_c_ were also evaluated and are shown in Figure 7a and b, respectively, as a function of the filler loading. Independently from the filler type, T_c_ increases significantly by 9–17 K in the composites. On first thought, this suggests that the fillers, in whole or in part, may act as crystallization nuclei. Then, CF_c_ is, in general, low, 1–2%, which, combined with the elevated T_c_, suggests an altered semicrystalline morphology. For example, we could expect that in the composites during melt crystallization, more crystals but ones of quite smaller sizes are formed. In the extreme cases of 20 and 30% hybrid, the high T_c_ values are accompanied by a high CF_c_ of ~28 and ~15%, respectively. We suspect that for these cases, the role of CNTs is quite strong in facilitating the crystallinity [28].

PLA is known to slowly and weakly crystallize, in general, compared with other semicrystalline polymers (polyethylene, polycaprolactone, etc.). This is evidenced, among other indicators, by the difficulty to crystallize during cooling and the evolution of crystallization also upon heating (cold crystallization, Figure 3a and Figure 6b). The clearest view for cold crystallization is that shown in Figure 3a, as it involves the prior suppression of melt crystallization and the subjection of the sample to strong supercooling by fast cooling. The corresponding temperature of cold crystallization, T_cc_, (Figure 7a) increases for low and moderate filler loadings and decreases for higher ones. In terms of the nucleation effects of lignin and hybrid, the results are compatible with those for the melt crystallization only for the high filler loadings. The evolution of cold crystallization is also connected with the mobility of the polymer chains, i.e., reflected on the T_g_. In this context, the low-filled composites exhibit the highest T_g_ (Figure 4a); thus, they exhibit slower diffusion of chains and, essentially, retarded development of cold crystallization, compared to the faster cold crystallization of the highly filled composites.

The melting temperature, T_m_ (Table 1), lying between 174 and 176 °C, demonstrates minor changes. Due to that, we expect insignificant changes in the quality of the formed crystals (size, density of the lamellae packing, etc.).

Finally, we should comment on the effects of crystallization on the glass transition. From Table 1 and in Figure 6c, we may conclude that the presence of the crystals is significant only when the CF_c_ is worth noting. This happens for the cases of 10–30 wt% hybrid, where the effects are quite strong concerning the strength of the glass transition (Δc_p_) rather than the T_g_, which barely changes.

### 3.4. Electrical Conductivity Aspects

**Figure 8:**
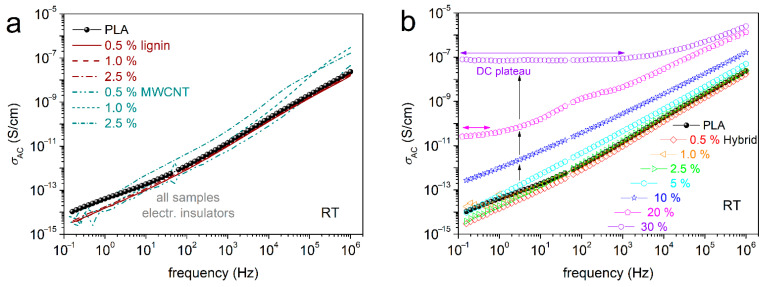
Comparative isothermal spectra of the real part of electrical conductivity (in AC), σ_AC_, as a function frequency at RT~20 °C for (**a**) PLA, PLA/lignin and PLA/MWCNTs and (**b**) PLA and PLA/hybrid lignin–CNTs.

BDS enables the evaluation of electrical conductivity, σ_AC_, via the measurement of the dielectric permittivity function (Equation (1), [59]). As expected, the neat PLA and the PLA/lignin composites exhibit electrical insulating character. This is manifested in Figure 8, as the σ_AC_(f) in the recorded spectra at room temperature (RT < T_g_) is, in general, low and σ_AC_ is frequency dependent. In addition, the PLA/CNT systems are also electrical insulators, even at the higher CNT loading of 2.5%.

We recall that the composites have been prepared by simple methods, without the employment of any surface modifications for CNTs. In previous works on CNT-filled polymer nanocomposites (including PLA) involving simple mixing routes, the electrons percolation threshold, p_c_ [36], has been found to be relatively high, e.g., between 5 and 6 wt%. This is in agreement with the discussed microscopic view for these composites (Figure 2). It is worth noting, on the other hand, that when more specific dispersion methodologies and CNT-surface modifications are involved, p_c_ has been evaluated at quite low CNT fractions (0.06–0.7%) [37,78] or even extremely low ones (0.0025%) [38].

Coming back to our composites, the addition of hybrid in PLA induces the following impact on conductivity. In Figure 8b, for up to 5% hybrid, the composites preserve the insulating behavior. For 10% hybrid, an overall σ_AC_ increase within the whole f range is recorded. Then, for gradually increasing the hybrid loading to 20 and 30%, σ_AC_ exhibits a stronger rise, by orders of magnitude. Interestingly, the increase is accompanied by the formation of a plateau. The latter suggests the independence of σ from f, at least for the lower frequencies. Such a plateau in polymers, with absolute σ values from ~10^−8^ to ~10^−6^ S/cm, usually denotes the ions transport (ionic conductivity) throughout the rubbery (liquid state) of the polymer matrix. This is not the case here, as PLA at RT is still in the glassy state and any ion transport is precluded. The plateau is recorded at both lower and higher temperatures. Therefore, we conclude that this plateau, else called the DC plateau, is due to the transport of electrons throughout the composites’ volume. Obviously, this is the case for the formation of continuous (percolating) electrons paths via CNTs [36].

**Figure 9:**
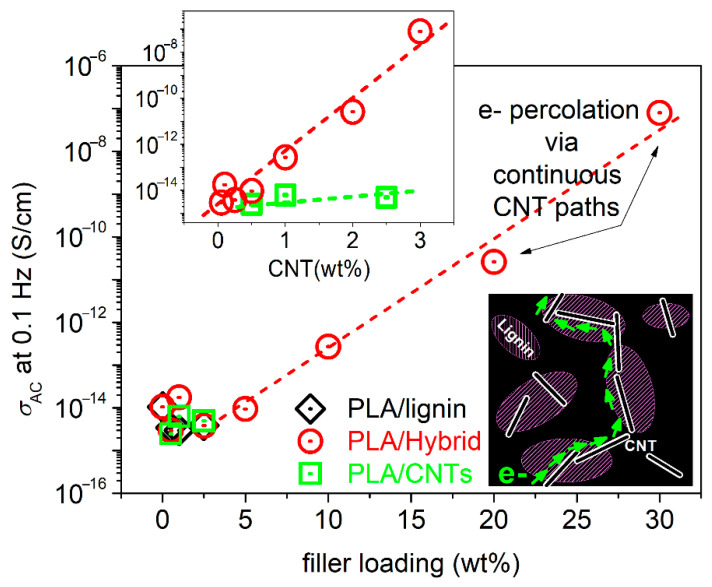
The filler wt% content dependence of the absolute conductivity, σ_AC_, values at the lowest frequency of recording, for PLA/lignin, PLA/hybrid and PLA/CNT. The inset graph shows the respective dependence from σ′ directly from the CNT loading. The added scheme presents the estimated mechanism of electronic conductivity via percolating CNT paths.

The results on the electrical conductivity can be summarized for all samples in Figure 9, wherein we have plotted the values for σ_AC_ at the lowest f of recording, i.e., 100 mHz, against the filler loading, for PLA/lignin, PLA/hybrid and PLA/CNTs. In the main figure, we obtain an almost linear dependence of conductivity, with no exceptional behavior among the different filler types.

Nevertheless, exploiting the knowledge that the unique free electron carriers here are the CNTs, in the inset to Figure 9, we plotted the CNT dependence of σ_AC_. We observe a striking effect here. The same amount of CNTs imposes undetectable effects in conductivity when being mixed with PLA, whereas it introduces significant electron conduction when being introduced to PLA via the hybrid lignin–CNT entities. Most probably, this is an interesting case of a synergistic [79] CNT–lignin effect, in the sense of favoring the CNT–CNT percolation (schematic in Figure 9). The said effect should not be far from the reality, as recalling the SEM images of Figure 2, the CNTs were found to be concentrated within the lignin-rich areas. Within these domains, the possibility for CNT percolation is larger, as compared to the case of mixing PLA with neat CNTs. Obviously, this is a strong speculation and needs further experimental manifestation.

Apart from the interest from the physics point of view, the result of enhancing the electrical conductivity here for relatively low CNT loadings within a mostly green polymer composite is quite interesting and promising, envisaging certain applications (e.g., conducting printing inks, etc.).

We should mention, from the methodological point of view, that a ‘precise’ evaluation of p_c_ for our systems from Figure 9 employing the percolation theory is precluded, due to the relatively few points of the actually conductive composites. In addition to that, the almost linear trend of σ_DC_ against the filler loading is not expected to produce a realistic/representative p_c_ value.

### 3.5. Segmental Dynamics

In the last section of this study, we investigate the segmental dynamics. This is achieved by following the formation of dielectric loss, ε″(f), peaks [80,81], which can be carried out only for the systems exhibiting an electronically insulating character. Therefore, PLA filled with 20 and 30% hybrid, which is electrically conductive, does not allow for such investigation. We also recall that for assessing the direct filler loading effects, the BDS measurements initiate with the polymers being in the amorphous state.

**Figure 10:**
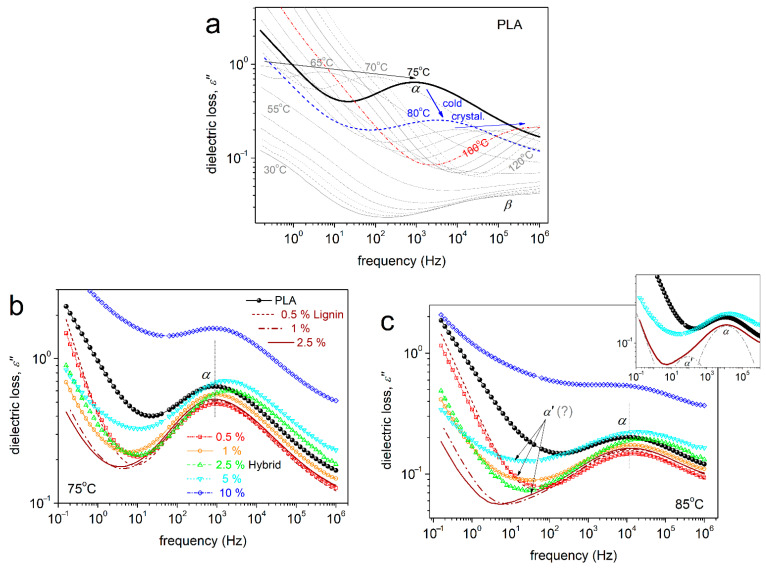
(**a**) Raw BDS data in the form of frequency and temperature dependence of the imaginary part of dielectric permittivity for neat PLA. Comparative ε″(f) for all samples at (**b**) 75 °C and (**c**) 85 °C. Indicated at selected temperatures are the recorded relaxation mechanisms (β, α, α′) as peaks and (**a**) the evolution of cold crystallization during the measurement.

The segmental dynamics can be monitored by the dipolar relaxation of the dipole moments being perpendicular to the polymer chain backbone [59]. The dipoles exhibit ‘recordable’ relaxation times, τ_rel_, for temperatures around T_g_ and higher. For such temperatures, the dielectric losses demonstrate the so-called α relaxation/process peak. The latter can be seen in Figure 10 (raw data) for selected systems and temperatures. With the temperature increasing gradually, the peaks migrate toward higher frequencies, as the polymer chains become more mobile. Thus, the dipolar τ_rel_ become gradually shorter. This is actually the reason for such a result to be referred to as ‘dynamics’ and the α relaxation being considered as the dielectric and dynamic analogue of the calorimetric glass transition. At temperatures above 85–90 °C, the amorphous polymer character gradually changes, as cold crystallization takes place. Please compare Figure 10a (BDS, blue arrow) with Figure 3a (DSC).

At the higher frequency side of Figure 10a and for temperatures below and close to T_g_, a weak peak addressed as β is recorded. This peak is due to the relaxation of local groups in PLA (-C=O on the chain backbone, [80]), which is not studied here. Additional phenomena being recorded within the temperature range of the measurements are related to ionic conductivity and interfacial polarizations [59]. The ionic conductivity is recorded as a sharp linear-like increase in the ε″ at the lower frequency side of the spectra and for T >> T_g_. Within similar frequency/temperature ranges, the interfacial polarization phenomena are recorded as strong peaks (not discussed further here).

Since the focus here is on the segmental relaxation, and in order to extract as much information as possible from the BDS data, we performed fitting [59] of ε″(f,T) by widely adopted mathematical model functions. The Havriliak–Negami (HN) function [59], described by Equation (3), was employed to fit the asymmetric peaks, in our case, the amorphous α relaxation.

(3) $$\varepsilon^{*}\left(f\right)=\frac{\Delta \varepsilon }{{\left[1+{\left(\frac{if}{f_{0}}\right)}^{\alpha_{HN}}\right]}^{\beta_{HN}}}+\varepsilon_{\infty }$$

In the HN function, *f*_0_ is a characteristic frequency related to the frequency of maximum dielectric loss, *ε*_∞_ is a value of the real part of the dielectric permittivity (ε′) at *f* >> *f*_0_, *α_HN_* is a shape parameter that denotes the width of relaxations times range and *β_HN_* is another shape parameter that evaluates the symmetry of the ε″(f) peak. *Δε* is the dielectric strength (~magnitude of the peaks) or else the corresponding contribution of the dipoles to the dielectric permittivity. The same model can be fitted for symmetric peaks, if needed, and this is achieved by fixing the asymmetricity parameter *β_HN_* as 1. This way, the HN models becomes the so-called Cole–Cole function [59]. To fit the ionic conductivity contribution, a specific linear term is used, with a slope equal to ~1 [59].

**Figure 11:**
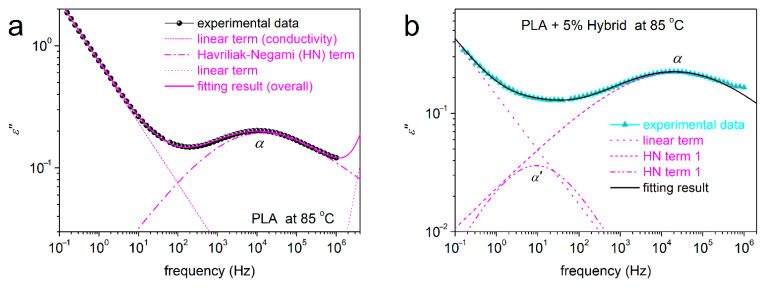
Examples of analysis of ε″(f) in terms of model functions, namely, selected for (**a**) neat PLA and (**b**) PLA + 5 wt% hybrid, at 85 °C.

The fitting process was ‘critically’ employed for all samples and two examples are demonstrated in Figure 11a for neat PLA and in Figure 11b for PLA + 5% hybrid. The fitting method was the same for the rest of the samples.

Interestingly, the critical fitting was quite fruitful in the sense that for all PLA/hybrid and PLA + 1 and 2.5% lignin composites, an additional relaxation could be resolved. This process, named as α′ (e.g., in Figure 10b and Figure 11b), was necessary to sufficiently fit the overall complex spectra. Actually, this an example of the power of analysis in BDS as an additional investigation tool [68,69,73,82]. In all cases and compared to α, the α′ relaxation was recorded at lower frequencies. This suggests slower/retarded mobility. In addition, α′ relaxation exhibits significantly lower strength (magnitude). From the models/shape point of view, α could be fitted only with the asymmetric HN function (α_HN_~0.5–0.6, β_HN_~0.6), whereas α′ was fitted by the symmetric Cole–Cole function (α_HN_~0.6, β_HN_~1).

**Figure 12:**
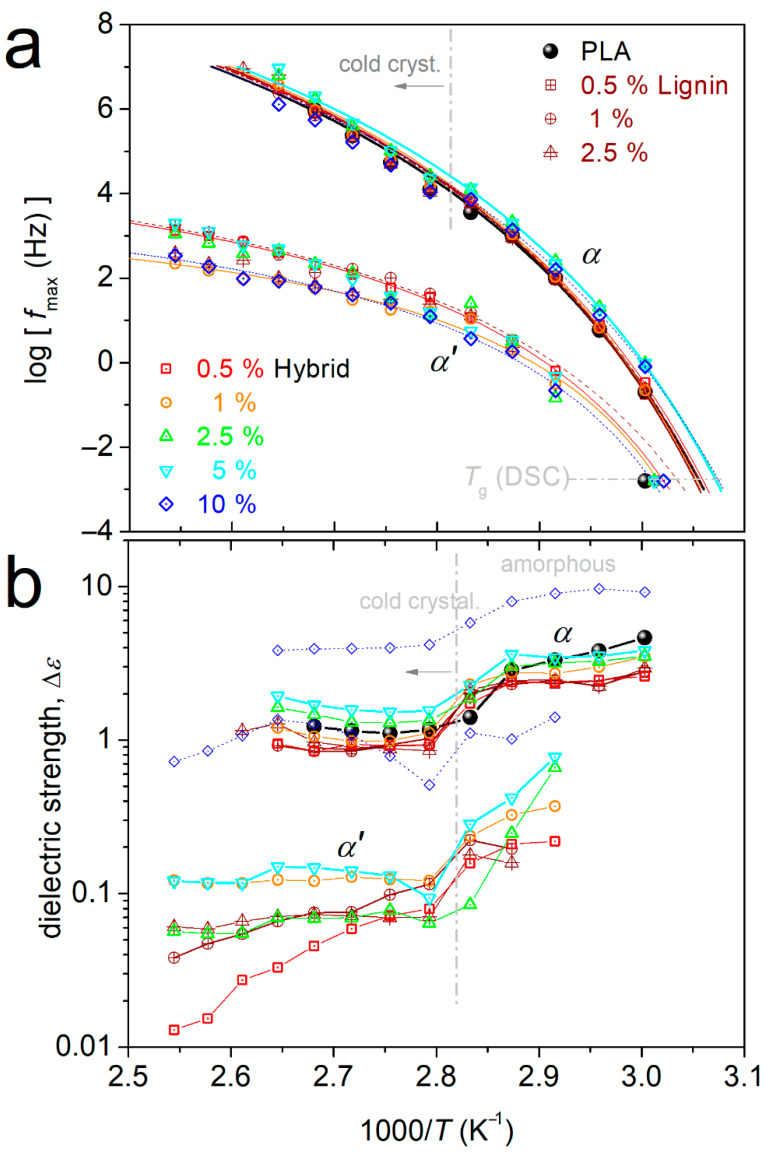
Dielectric relaxation map in terms of (**a**) Arrhenius diagrams for the time scale of all samples and relaxation and (**b**) the corresponding reciprocal temperature dependence of the dielectric strength. Added in (**a**) are the corresponding calorimetric values for T_g_, while the added lines crossing the dielectric data are fittings of the Vogel–Tammann–Fulcher–Hesse equation on the points of α and α′ relaxation prior to the interference of cold crystallization.

From the fitting results, we were able to construct the so-called molecular dynamics map in terms of the reciprocal temperature, 1000/T, and dependence of the relaxation frequency maxima, f_max_, for all relaxations. This is presented in Figure 12a, wherein, along with the dielectric data, we have added, for comparison, the calorimetric points for the glass transition temperature. In Figure 12b, we present the 1000/T dependence of Δε, at the same temperature range.

In Figure 12b, α relaxation in neat PLA exhibits a ‘curved’ time scale, which is in accordance with previous studies on PLA [21,80,81] (and references therein). The curved time scale is characteristic for bulk cooperative dynamics. The trend can be mathematically expressed by the so-called Vogel–Fulcher–Tammann–Hesse (VFTH) equation (Equation (4)) [59,83].

(4) $$f(T)=f_{0,VFTH}\cdot e^{-\frac{DT_{0}}{T-T_{0}}}$$

In Equation (3), *f*_0,*VFTH*_ is a frequency constant and *T*_0_ is the Vogel temperature at which *f*_0,*VFTH*_ → 0. *D* is the so-called fragility strength parameter [83]. The VFTH model was well fitted to our results on α and on α′. In particular, for α, the VFTH fitting was successful by fixing *f*_0,*VFTH*_ to the phonon frequency of 10^13^ Hz [83], whereas this was not valid for α′.

The time scale of α for PLA seems to not significantly change in the composites. However, we may observe a weak tendency for the acceleration of α for the larger loadings of hybrid. The result is quite similar to the effects recorded on the calorimetric T_g_ (points in Figure 12a). The dielectric strength does not exhibit systematic alternations in the presence of the fillers, with the exception of PLA + 10% hybrid, wherein an overall significant Δε rise is recorded. This is, most probably, connected with the overall permittivity/conductivity rise for this sample, due to the presence of the highly conducting CNTs at a generally high amount (please compare with Figure 8b). Therefore, it is not essential to assign the increased Δε to stronger dipolar contribution (chain mobility).

From the time scale data on α, we may estimate the *D* parameter (Equation (4)) and, via the latter, calculate the fragility index for the segmental relaxation, *m_α_*. The calculation is performed employing Equation (5) [21,83].

(5) $$m_{\alpha }=16+590/D$$

Additionally, the dielectric glass transition temperature can be evaluated by extrapolating the VFTH fits to the equivalent frequency of conventional DSC at T_g_, f_eq,DSC_, i.e., when the chains’ relaxation time becomes 100 s or else at f_eq,DSC_~10^−2.8^ Hz.

**Figure 13:**
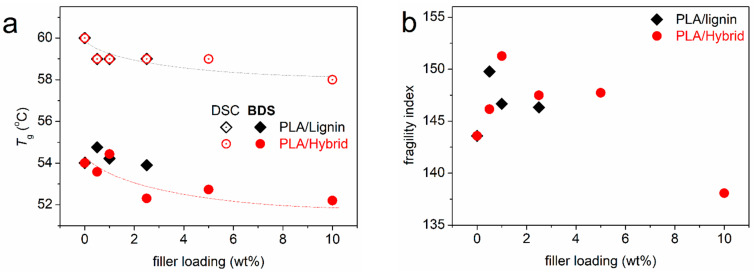
The filler content dependences of (**a**) the dielectric and calorimetric T_g_s and (**b**) the fragility indices of α relaxation, for all systems. The added lines are used as guides for the eye.

The T_g,diel_ is shown comparatively with the calorimetric T_g_, as a function of the filler loading in Figure 13a. The generally decreasing trends suggest that in the composites, there is a weak acceleration of the bulk-like polymer chains’ diffusion. On the other hand, in Figure 13b, m_α_ (143 in PLA) exhibits a moderate increase in the composites (146–152), with the exception of the extreme case of PLA + 10% hybrid, where m_α_ is quite lower (138). The increased fragility, at least for simple polymer systems, can be discussed in terms of increased cooperativity levels and, equivalently, in terms of the shortening of the nanometric cooperativity length, ξ [84,85]. We will come back to this point later.

We now come to the interesting case of α′ relaxation. The process exhibits a ‘cooperative character’, as manifested by the VFTH timescale in Figure 12a. Furthermore, upon its extrapolation to the calorimetric frequencies area in Figure 12a, the experimental data of α′ coincide quite well with the calorimetric T_g_, similarly to the case of α relaxation. Combining these with the absence of the process in pure PLA, we may conclude that this is a segmental-like process with retarded (modified) dynamics arising from the interfacial polymer–filler region. Such a relaxation was observed for the first time in polydimethylsiloxane/silica nanocomposites by Fragiadakis et al. [68] and, later, within various other nanocomposites [66,69,70,73,82]. Adopting the methodologies used in these previous studies, we can estimate the amount of the interfacial polymer with the modified dynamics, based on its dielectric strength (Δε_α_ and Δε_α′_, Figure 13b). We recall that in a previous section, we estimated the amount of the immobile interfacial polymer (RAF) based on its absent contribution to the calorimetric strength of the glass transition.

Thus, employing some assumptions, for example, equal polarizability between the bulky and the interfacial chain’ dipoles, we calculate the interfacial polymer fraction, IPF, by Equation (6) [66,69,70,73].

(6) $$\mathrm{IPF}=∆\varepsilon_{\alpha ΄}/\left(∆\varepsilon_{\alpha }+∆\varepsilon_{\alpha ΄}\right)$$

**Figure 14:**
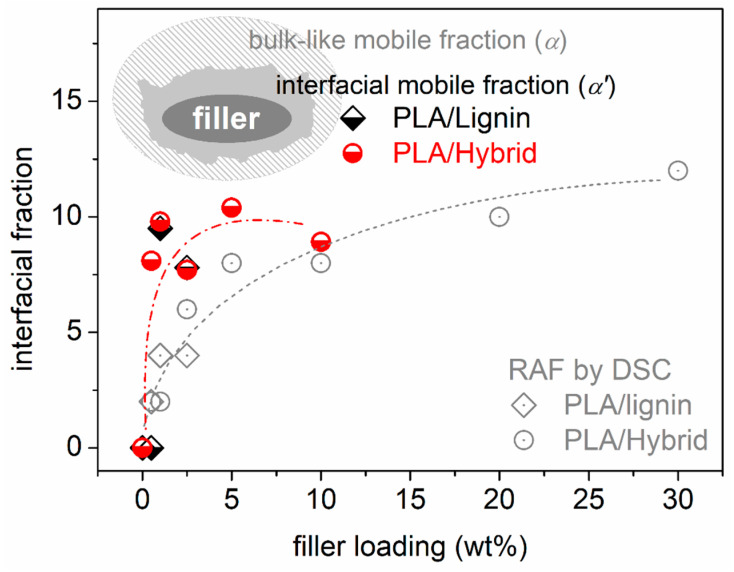
The interfacial polymer fraction as estimated from the dielectric and calorimetric data on segmental dynamics, being presented as a function of the filler wt% loading.

The IPF values have been plotted against the filler loading and are shown comparatively with RAF (by DSC). The IPF values lay between 0% (PLA) and 10% (5% hybrid) and, for a fixed filler loading, they are significantly larger than those of the RAF (0–8%). Possibly, the RAF and IPF have different physical origins or/and the IPF may include the RAF (inset scheme in Figure 14) [66]. This is actually still an open debate in the literature. The filler loading dependence of RAF increases monotonically, whereas that of IPF does not, as it sharply increases even for the lowest hybrid amounts and barely increases further. This is possibly related to the parallel drop in the quality of the hybrid particles dispersion [73].

It should be reported that the shape parameters for α and α’ relaxations (*α*_HN_ and *β*_HN_) do not demonstrate any systematic or interesting changes between the different systems. Within previous cases, the values of ‘*α*_HN_ × *β*_HN_’ have been used as representatives of the relaxation times width [86], in other words, the degree of homogeneity of the relaxation modes.

We come back to the recorded discrepancy between DSC and BDS regarding the T_g_ values (Figure 13a). Such or similar discrepancies have been recorded in the past within a variety of systems, especially when ‘complex’ polymeric phases are involved [57,87,88]. Next to the in-principle different techniques, a possible reason for the discrepancy has been proposed to originate from the existence of dynamical heterogeneities. Such heterogeneities are, for example, due to significant alternations in the cooperativity lengths, in particular, at different temperatures [89], being followed via different physical process (e.g., static and pure thermally in DSC, dielectrically and dynamically in BDS). Comparing with various nanocomposites studied by similar methods, Koutsoumpis et al. [88] concluded that the presence of an interfacial polymer may itself induce the said dynamical heterogeneities. This could be also the explanation in the present work.

Finally, combining all the information together, we may conclude with a unified model for rationalizing the overall situation. An amount of PLA chains is concentrated around the hybrid particles, exhibiting a modified mobility (retarded or vanished). On the other hand, it seems that away from the particles, the bulky polymer may exhibit a slightly lesser density that enhances the PLA chain diffusion/mobility. Similar results rationalized by such scenarios have been recently reported for polyester-based complex systems (including PLA) [22,24,57].

## 4. Conclusions

For the recently synthesized PLA/hybrid (lignin–CNT), envisaged for use in conducting printing inks, a series of new results by complementary methods were recorded. The main results gained by the extensive investigation on the structure–properties relationships are as follows:

- PLA and lignin are electrical insulators due to the absence of free electrons. The conducting character (DC conductivity plateau) of the composites was offered by the addition of >2% CNTs. Interestingly, this was found true only in the case of hybrid particles, and not by the addition of neat CNTs in PLA at similar amounts.
- The result suggests an additional effect of lignin in the sense of facilitating the formation of CNT continuous paths (electronic percolation), which is, interestingly, compatible with the recorded microscopic view by SEM.
- For the composites, we obtained proofs for the formation of an interfacial polymer layer around the particles, without the implementation of strong polymer–particles interactions.
- A mild drop in the T_g_ of PLA (60 °C), by 1–2 K, was recorded in the presence of the fillers, suggesting the formation of, in general, softer composites, compared with neat PLA.
- The latter is accompanied by the almost unchanged T_m_ of PLA, which remains relatively low (174–176 °C).

These effects imposed by the hybrid particles are actually wanted, considering, on one hand, the envisaged application in printing inks (conductivity) and, on the other hand, the processing in general. For example, we expect that the viscosity of the composites is lower than that of the unfilled PLA matrix. Among other indicators, this suggests a more beneficial processing with less energy consumption for the preparation and the application.

## Figures and Tables

**Table 1:** Samples under investigation and values of interest obtained by calorimetry: glass transition temperature, *T*_g_, normalized heat capacity change, Δ*c*_p,norm_, calculated rigid amorphous fraction, RAF, in the amorphous state, low temperature cold crystallization temperature, *T*_cc1_, melt crystallization temperature and normalized enthalpy, *T*_c_ and Δ*H*_c,norm_, respectively, crystalline fraction estimated from the latter enthalpy, CF_c_, and melting temperature, *T*_m_.

Sample	Lignin (wt%)	CNT (wt%)	DSC (Fast Cooled, Amorphous)				DSC (Cooled at 20 K/min)				
			*T*_g_ (°C)	Δ*c*_p,norm_ (J/g_PLA_∙K)	RAF (%)	*T*_cc1_ (°C)	*T*_c_ (°C)	Δ*H*_c,norm_ (J/g_PLA_)	CF_c_ (%)	*T*_g_ (°C)	*T*_m_ (°C)
PLA (neat)	0	0	60	0.50	0	97	80	1.3	1	60	175
PLA + 0.5% lignin	0.5	0	59	0.49	2	102	92	2.3	2	59	176
PLA + 1.0% lignin	1.0	0	59	0.48	4	104	90	1.0	1	59	176
PLA + 2.5% lignin	2.5	0	59	0.48	4	104	89	1.8	2	59	176
PLA + 0.5% hybrid	0.45	0.05	59	0.49	2	102	92	1.5	2	59	176
PLA + 1.0% hybrid	0.9	0.1	59	0.49	2	103	91	1.3	1	59	175
PLA + 2.5% hybrid	2.25	0.25	59	0.47	6	105	92	1.8	2	58	175
PLA + 5.0% hybrid	4.5	0.5	59	0.46	8	105	89	1.4	2	59	175
PLA + 10.0% hybrid	9	1	58	0.46	8	104	92	2.0	2	61	174
PLA + 20.0% hybrid	18	2	58	0.45	10	99	97	26	28	57	174
PLA + 30.0% hybrid	27	3	58	0.44	12	100	94	14	15	57	174

## Data Availability

The DSC, FTIR, BDS and conductivity raw data are available on Zenodo (doi: https://doi.org/10.5281/zenodo.15281532). The rest of the data supporting this article will be available upon request to the corresponding authors, uniquely in the frame of private communication.
