# Correction: Klonos et al. Segmental Mobility, Interfacial Polymer, Crystallization and Conductivity Study in Polylactides Filled with Hybrid Lignin-CNT Particles. *Nanomaterials* 2025, *15*, 660

**DOI:** 10.3390/nano15201599

**Published:** 2025-10-21

**Authors:** Panagiotis A. Klonos, Rafail O. Ioannidis, Andreas Pitsavas, Nikolaos D. Bikiaris, Sofia P. Makri, Stefania Koutsourea, Alexios Grigoropoulos, Ioanna Deligkiozi, Alexandros Zoikis-Karathanasis, Apostolos Kyritsis, Dimitrios N. Bikiaris

**Affiliations:** 1 Dielectrics Research Group, Department of Physics, National Technical University of Athens, Zografou Campus, GR-15780 Athens, Greece; akyrits@central.ntua.gr; 2 Laboratory of Polymer Chemistry and Technology, Department of Chemistry, Aristotle University of Thessaloniki, GR-54124 Thessaloniki, Greece; rafailio@chem.auth.gr (R.O.I.); apitsava@chem.auth.gr (A.P.); nikompik@pharm.auth.gr (N.D.B.); dbic@chem.auth.gr (D.N.B.); 3 Creative Nano PC, 43 Tatoiou, Metamorfosi, GR-14451 Athens, Greece; s.makri@creativenano.gr (S.P.M.); s.koutsourea@creativenano.gr (S.K.); a.grigoropoulos@creativenano.gr (A.G.); a.karathanasis@creativenano.gr (A.Z.-K.); 4 AXIA Innovation GmbH, Fritz-Hommel-Weg 4, 80805 München, Germany; ide@axia-innovation.com

In the original publication [1], reference 7, “Oladapo, B.I.; Olawumi, M.A.; Olugbade, T.O.; Tin, T.T. Advancing sustainable materials in a circular economy for decarbonisation. *J. Environ. Manag.* **2024**, *360*, 121116”, was retracted prior to this publication, and the authors would like to replace it with the following reference:

Bikiaris, N.D.; Koumentakou, I.; Samiotaki, C.; Meimaroglou, D.; Varytimidou, D.; Karatza, A.; Kalantzis, Z.; Roussou, M.; Bikiaris, R.D.; Papageorgiou, G.Z. Recent Advances in the Investigation of Poly(lactic acid) (PLA) Nanocomposites: Incorporation of Various Nanofillers and their Properties and Applications. *Polymers* **2023**, *15*, 1196. 

The authors state that the scientific conclusions are unaffected. This correction was approved by the Academic Editor. The original publication has also been updated.
